# Successful Left Bundle Branch Area Pacing Using the Sheath‐In‐Sheath Technique in a Patient With Right Atrial Enlargement and Tricuspid Annuloplasty Ring

**DOI:** 10.1002/joa3.70180

**Published:** 2025-08-21

**Authors:** Hiroyuki Kato, Taku Sakurai, Jun Sato, Kazumasa Suga, Hisashi Murakami

**Affiliations:** ^1^ Department of Cardiology Japan Community Health Care Organization Chukyo Hospital Nagoya Japan; ^2^ Department of Pediatric Cardiology, Chukyo Children's Heart Center Japan Community Health Care Organization Chukyo Hospital Nagoya Japan

**Keywords:** conduction system pacing, left bundle branch pacing, sheath‐in‐sheath technique, tetralogy of Fallot, tricuspid annuloplasty

## Abstract

We report a successful LBBAP lead implantation using the sheath‐in‐sheath technique in a patient with RA enlargement and a TAP ring. The sheath‐in‐sheath technique can serve as a valuable alternative approach in patients with structural heart abnormalities in whom LBBAP lead placement with a standard delivery system is technically challenging.

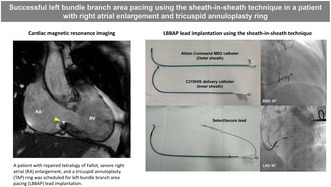

A 55‐year‐old woman was admitted to our hospital with sinus node dysfunction and presyncope symptoms (Figure 1A,B). She was diagnosed with tetralogy of Fallot at birth and underwent transannular patch repair at 4 years of age. At 49 years of age, she underwent two sessions of radiofrequency catheter ablation for atrial tachycardia originating from the right atrium (RA). Electroanatomical three‐dimensional mapping during the procedure revealed severe RA dilatation and extensive low‐voltage areas. During follow‐up, she developed severe tricuspid regurgitation and right ventricular (RV) enlargement. At 53 years of age, she underwent pulmonary valve replacement using an Inspiris 25‐mm valve (Edwards Lifesciences, Irvine, CA, USA) and tricuspid annuloplasty (TAP) with a Tri‐Ad Adams 30‐mm ring (Medtronic, Minneapolis, MN, USA). Cardiac magnetic resonance imaging performed prior to pacemaker implantation revealed enlargement of the RA and RV, as well as a surgically constricted tricuspid annulus with the TAP ring in place (Figure 1C). The left ventricular ejection fraction (LVEF) was 50%. In accordance with the current guideline‐directed therapy, transvenous dual‐chamber pacemaker implantation was scheduled to address symptomatic sinus node dysfunction [1]. Although the indication for left bundle branch area pacing (LBBAP) in patients with sinus node dysfunction and preserved LVEF remains controversial [2], informed consent was obtained from the patient for the implantation of a ventricular lead in the left bundle branch area, based on the following considerations: the presence of extensive low‐voltage areas in the RA suggested a potentially high risk of developing atrioventricular block due to intra‐atrial conduction disturbances and refractory atrial arrhythmias requiring atrioventricular nodal ablation.

**FIGURE 1:**
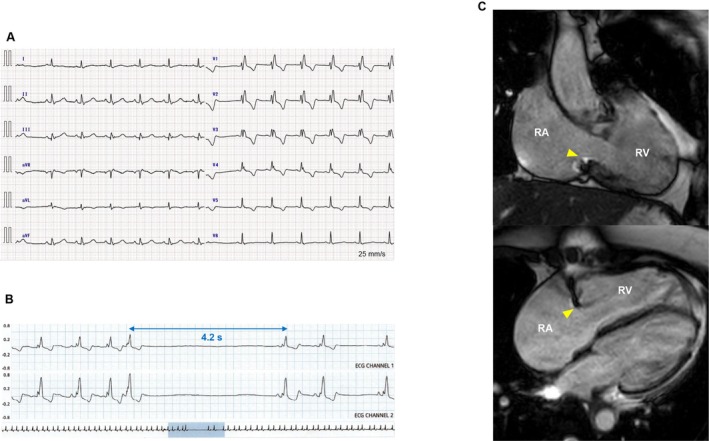
(A) Preoperative 12‐lead electrocardiogram during sinus rhythm, showing right bundle branch block with a QRS duration of 160 ms. (B) Holter electrocardiogram at the moment of presyncope, showing a 4.2‐s prolongation of the RR interval following an atrial premature contraction. (C) Cardiac magnetic resonance images in right anterior oblique (*upper panel*) and four‐chamber (*lower panel*) views, showing enlargement of the RA and RV. The *yellow arrowhead* indicates a surgically constricted tricuspid annulus with a tricuspid annuloplasty ring. RA, right atrium; RV, right ventricle.

The device implantation was performed under local anesthesia. A generator pocket was first created in the left infraclavicular area, followed by the insertion of 7‐Fr peel‐away sheaths via the axillary vein using an extrathoracic puncture technique. LBBAP lead implantation was performed using a C315HIS delivery catheter (Medtronic) and a lumenless SelectSecure lead (model 3830‐69 cm; Medtronic) [3]. Initially, we attempted to insert the delivery catheter into the RV by advancing the guidewire into the pulmonary artery. However, the enlarged RA caused the delivery catheter to deviate inferior to the tricuspid valve, and the catheter tip was obstructed by the TAP ring, which prevented its insertion into the RV. Therefore, we employed a sheath‐in‐sheath technique: a stiffer delivery catheter for coronary sinus lead placement (Attain Command MB2; Medtronic) was first advanced into the RV to serve as the outer sheath. The C315HIS delivery catheter was then introduced into it as the inner sheath. The preparation procedure for the sheath‐in‐sheath technique is illustrated in Figure 2.

**FIGURE 2:**
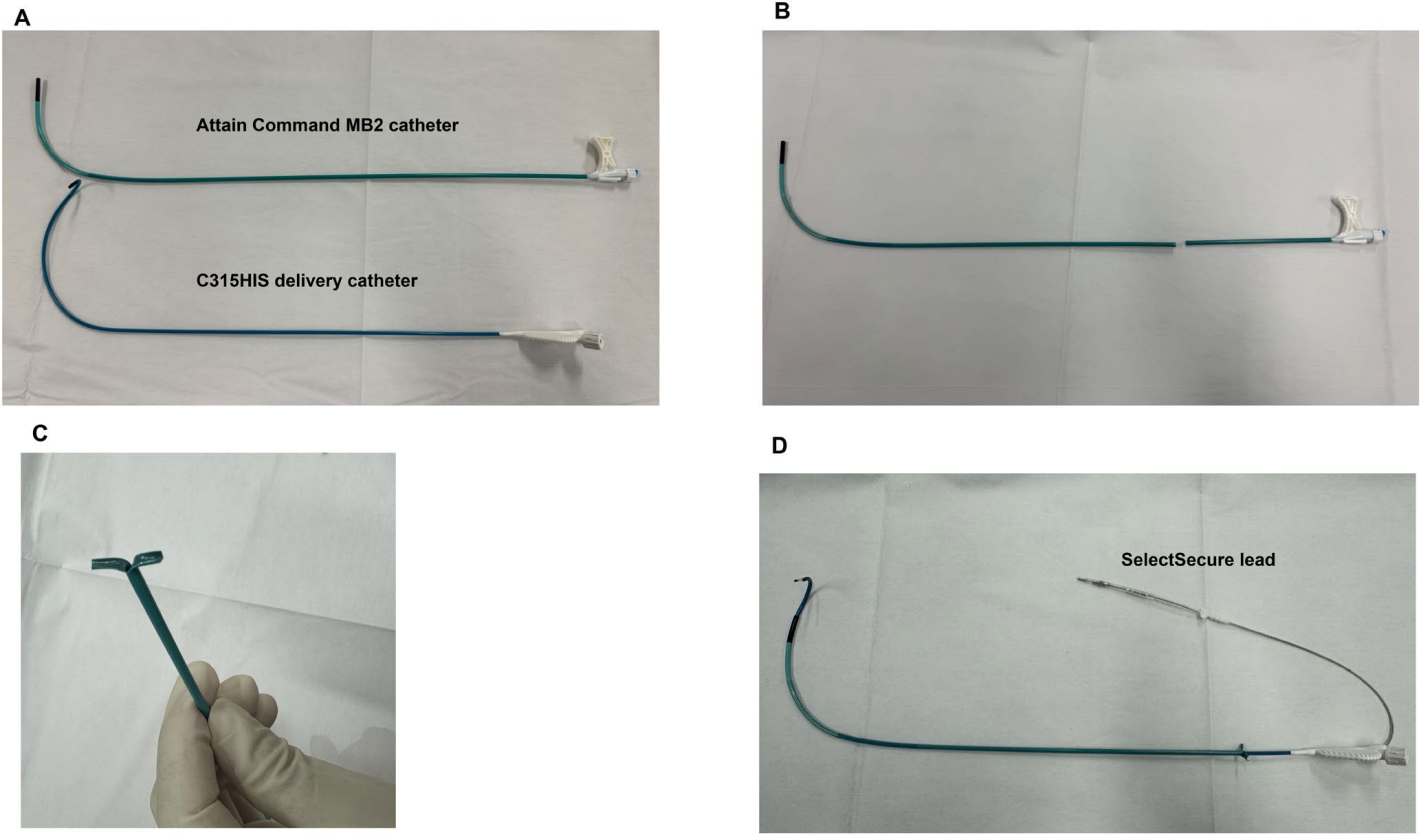
Preparation for the sheath‐in‐sheath technique. (A) Attain Command MB2 coronary sinus lead delivery catheter for the outer sheath and C315HIS delivery catheter for the inner sheath. (B) It is necessary to trim 10–15 cm from the proximal end of the outer sheath, as the inner sheath is shorter than the outer sheath. (C) A longitudinal cut is made in the proximal portion of the outer sheath and folded back. This serves the following purposes: to provide a precut that facilitates sheath slitting, to prevent full insertion of the outer sheath into the vessel, and to allow the sheath to be easily grasped with forceps during slitting. (D) Sheath‐in‐sheath configuration with an Attain Command MB2 catheter (outer sheath) and a C315HIS catheter (inner sheath), with a Select Secure lead inserted.

After replacing the 7‐Fr peel‐away sheath with a 9‐Fr sheath, the outer sheath was advanced over the guidewire and successfully passed through the RV (Figure 3A,B). The C315HIS delivery catheter was then positioned approximately 15 mm toward the RV apex from the His‐bundle area through the outer sheath (Figure 3C). Subsequently, the lead was deployed deep into the interventricular septum, resulting in successful left bundle branch capture (Figures 3D and 4). As satisfactory lead parameters were obtained (capture threshold, 0.5 V at 0.4 ms; R‐wave amplitude, 10.0 mV; pacing impedance, 665 Ω), the C315HIS delivery catheter was removed first, followed by careful removal of the outer sheath. Due to the widespread low‐voltage areas in the RA, an atrial lead (SelectSecure lead, model 3830‐69 cm; Medtronic) was implanted using a C315HIS delivery catheter at the posterior atrial septum, where voltage amplitude was relatively preserved. Both atrial and ventricular leads were connected to a pacemaker generator (Azure XT DR; Medtronic). The final lead positions are presented in Figure 3E. The patient experienced no postoperative complications, and her clinical symptoms improved. During the 2‐month follow‐up, atrial and ventricular pacing thresholds remained stable at 1.0 V and 0.5 V, respectively, with no evidence of conduction system capture loss.

**FIGURE 3:**
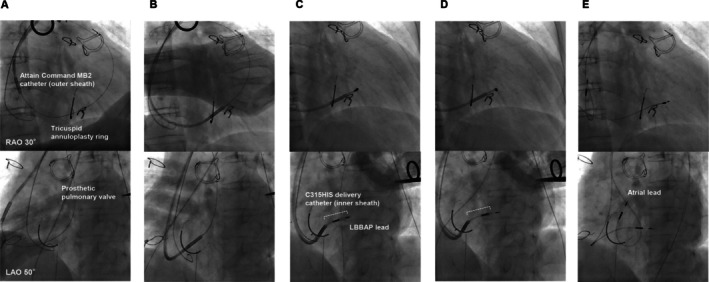
Fluoroscopic images during LBBAP lead implantation using the sheath‐in‐sheath technique. (A) The guidewire was positioned deep into the pulmonary artery. Note that the outer sheath deviated away from the tricuspid valve toward the dilated RA free wall. (B) The tip of the outer sheath was successfully advanced into the RV over the guidewire. (C) The inner sheath was positioned at the septum approximately 15 mm apical to the His‐bundle region through the outer sheath, and the LBBAP lead helix was positioned in contact with the septal surface. The *dotted white line* indicates the portion of the inner sheath protruding from the outer sheath. (D) Owing to adequate backup support, the LBBAP lead was advanced relatively easily into the deep septum. (E) Final lead position. The atrial lead was positioned in the posterior atrial septum. LAO, left anterior oblique; LBBAP, left bundle branch area pacing; RA, right atrium; RAO, right anterior oblique; RV, right ventricle.

**FIGURE 4:**
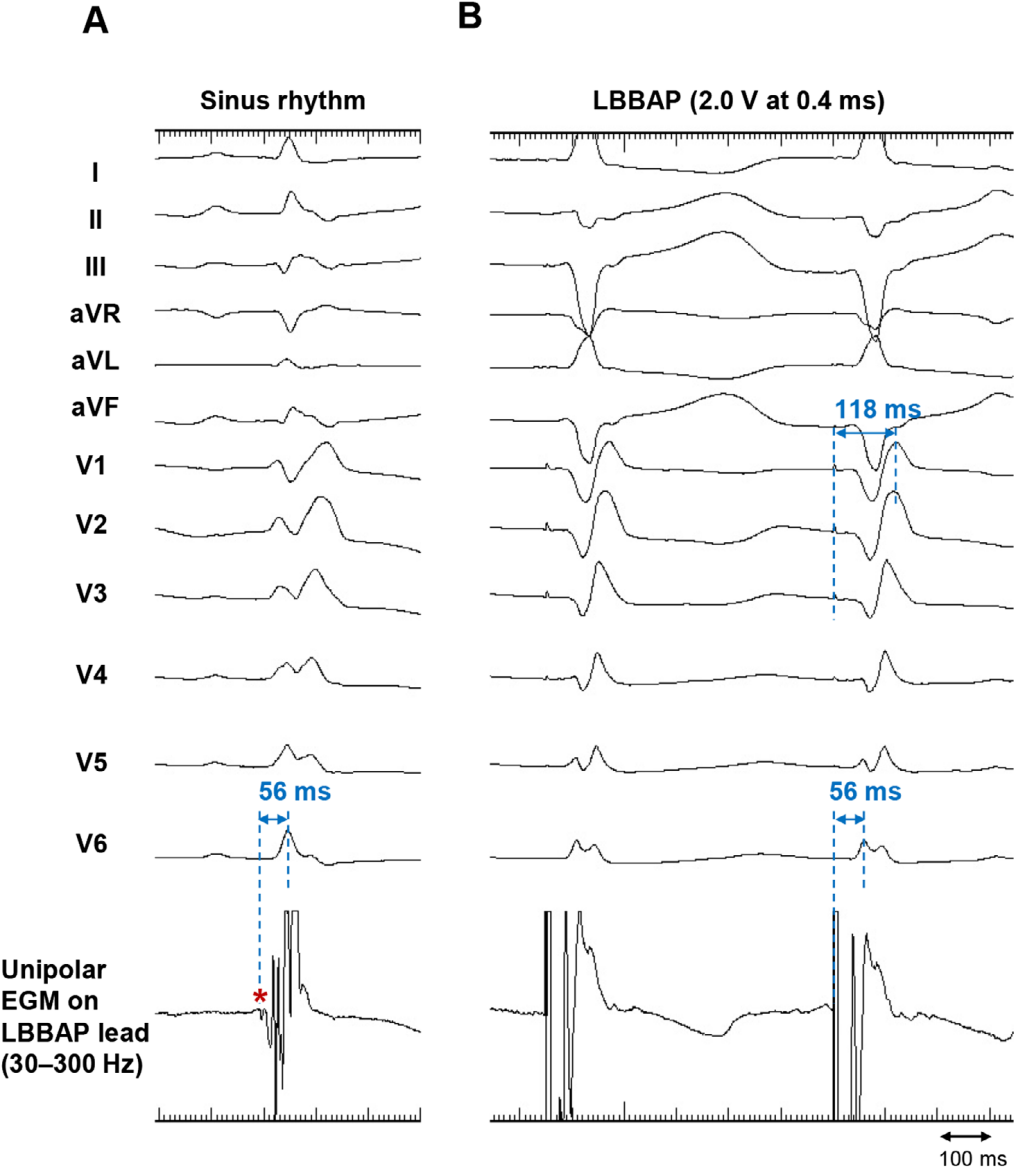
Twelve‐lead electrocardiogram and unipolar electrogram on LBBAP lead after deep septal deployment. (A) During sinus rhythm, a left bundle branch (LBB) potential preceding the QRS complex was confirmed (*red asterisk*). The interval from the LBB potential to the R‐wave peak in lead V6 (LBBpo‐V6 RWPT) was 56 ms. (B) During left bundle branch area pacing (LBBAP) at 2.0 V at 0.4 ms, the interval from the pacing spike to the R‐wave peak in lead V6 (V6 RWPT) was 56 ms, which was identical to the LBBpo‐V6 RWPT. The V1 RWPT was 118 ms, and the V6‐V1 interpeak interval was 62 ms. The QRS duration during LBBAP was 135 ms.

LBBAP lead implantation can be particularly challenging in structurally abnormal hearts, such as those with chamber enlargement, congenital heart disease, or a hypertrophied interventricular septum [3, 4]. This challenge often stems from limitations associated with the delivery catheter, including difficulties in reaching the target site, insufficient catheter length, and inadequate backup support for deep septal lead deployment. To address these challenges, the sheath‐in‐sheath technique is considered a potentially viable solution to facilitate a successful LBBAP in such complex conditions [4, 5]. In our case, RA enlargement and the presence of a TAP ring obstructed the passage of the C315HIS delivery catheter across the tricuspid valve. However, by employing the sheath‐in‐sheath technique, successful entry into the RV was achieved using a stiffer and more linear CS lead delivery catheter as the outer sheath. Furthermore, the sheath‐in‐sheath technique allowed the C315HIS delivery catheter to establish firm contact with the septal surface, providing sufficient backup support and enabling relatively easy deployment of the deep septal lead into the interventricular septum. Vijayaraman and Ellenbogen proposed that in the sheath‐in‐sheath technique, the outer sheath is removed first, followed by the C315HIS sheath [6]. However, in our case, removing the outer sheath first was considered potentially challenging, as it could cause the C315HIS delivery catheter to shift toward the dilated RA free wall, which could lead to dislodgement of the LBBAP lead. Consequently, we decided to remove the C315HIS catheter, followed by the outer sheath. The slitting technique used for removing the residual outer sheath in this case is demonstrated in the Video S1.

In conclusion, this case highlights the usefulness of the sheath‐in‐sheath technique in achieving successful LBBAP lead implantation in a patient with severe RA enlargement and a TAP ring. The sheath‐in‐sheath technique can serve as a valuable alternative approach in patients with structural heart disease or abnormalities in whom LBBAP lead placement with a standard delivery system is technically challenging.

## Ethics Statement

This study was approved by the Ethics Committee of Chukyo Hospital. This study was conducted in accordance with the principles of the Declaration of Helsinki.

## Consent

The authors confirm that written consent for the submission and publication of this case report, including images and associated text, was obtained from the patient.

## Conflicts of Interest

The authors declare no conflicts of interest.

## Supporting information


**Video S1:** Slitting technique for the residual outer sheath. This video demonstrates the slitting technique for the outer sheath following removal of the C315HIS delivery catheter (inner sheath). First, the blade of an adjustable slitting cutter (Adjustable Slitter, model 6232ADJ; Medtronic) was inserted into the precut notch at the proximal end of the outer sheath to secure the lead within the cutter. The bent tip of the outer sheath was then firmly grasped with mosquito forceps and pulled back for complete removal.

## Data Availability

The data underlying this article will be shared upon reasonable request to the corresponding authors.
